# Editorial: Effects of Prenatal Opiate Exposure

**DOI:** 10.3389/fped.2019.00455

**Published:** 2019-11-07

**Authors:** Henrietta S. Bada, Eric W. Reynolds

**Affiliations:** ^1^Department of Pediatrics, University of Kentucky, Lexington, KY, United States; ^2^Department of Pediatrics, University of Texas Health Science Center at Houston, Houston, TX, United States

**Keywords:** neonatal abstinence syndrome (NAS), drug abuse and addiction, editorial, newborn, drug withdrawal symptoms

Non-prescription opioid abuse continues to to be a growing problem in the United States. According to the National Survey on Drug Use and Health (NSDUH)−2014: 4.3 million Americans engaged in non-medical use of prescription painkillers in the last month; ~1.9 million Americans met criteria for prescription painkiller use disorder based on their use in the past year; and, 1.4 million people used non-medical prescription painkillers for the first time in the past year (1). Opioid overdoses increased 30 percent from July 2016 through September 2017 in 52 areas in 45 states (2). It is estimated that everyday, more than 115 people in the United States die after overdosing on opioids (3). The smallest subjects afffected by this crisis are newborn infants suffering from Neonatal Abstinence Syndrome (NAS). It is estimated that between 50 and 94% of infants with *in utero* exposure to opiates will have postnatal manifestations of withdrawal (4, 5). The national incidence of NAS has increased from 3.4 to 5.8 per 1000 live births between 2009 and 2012 (6). Neonatal ICU admissions for NAS increased from 7/1000 live births in 2004 to 27/1000 in 2013 (7). However, these statistics obscure the regional variation in the distribution of NAS, with some units seeing very few patients per year while others are experiencing epidemic numbers of affected infants.

In this Research Topic issue of *Frontiers*, we bring together a diverse panel of scientific researchers to give historical perspectives, basic science work, clinical investigation, and a look at the future of NAS research. We would like to offer our thanks to *Frontiers* for the opportunity to bring you this topic and to the readers, for your interest in this topic, and the work of all contributors of the included authors.

## Historical Perspectives

Gomez-Pomar and Finnegan give a comprehensive history of the exploding opioid epidemic. They describe over 4000 years of drug history from opium use by ancient Sumerians, to the first pharmacologic marketing of morphine in 1827, to the use of synthetic opioids and addiction we see today. Parallel to the increase in opioid addiction has been the burgeoning number of NAS cases. They go on to describe the current state of diagnosis, assessment and management of NAS.

## Basic Science Research

The article by Hauser and Knapp provides a basic science perspective on how opioid drugs disrupt neuronal and glial maturation in the central nervous system. The authors describe the tendency for opioids to inhibit and delay growth of neurons and associated CNS cells in specific brain regions, providing a plausible explanation for the clinical presentation of infants with NAS and adults with addiction. Sithisarn et al. give an accounting of their experience with an animal model of *in utero* oxycodone exposure. They found rats with *in utero* oxycodone exposure exhibited excessive hyperactivity as adults. They did not find the same cognitive impairment we see in human studies of *in utero* opioid exposure, but this may be due to methodological issues (dose, timing of exposure). Thus, further work in this area is warranted.

## Clinical Investigations

Pandey et al. describes a case series of twins with prenatal substance exposure and their subsequent discordance or concordance of clinical presentation. Abu Jawdeh et al., describe their finding of preterm infants and respiratory control. Interestingly, although clinicians usually consider preterm infants to be not likely to display symptoms of NAS, the authors found substance exposed infants spent more time with lower SpO_2_ than unexposed infants, suggesting that these infants may be displaying symptoms not previously suspected to be NAS related. Similarly, the work by Reynolds et al., explores another previously undescribed area of physiology, looking at non-nutritive suck in NAS. The fact that swallow-breath coordination in infants affected by NAS is similar to preterm infants and not a separate pathologic entity, seems to be in line with the disruption of neuronal and glial maturation described by Hauser and Knapp. How or if these infants catch up to unaffected infants remains to be determined. The work by Subedi et al. may also be related to Hauser and Knapp's findings. They found that infants affected by NAS have elevated plasma brain-derived neurotrophic factor (BDNF). Given that prenatal opioid exposure disrupts neuronal and glial growth and maturation, it is not out of the question to hypothesize that BDNF levels may be upregulated in an effort to stimulate new growth and development in the area. Further investigation is needed. Palla et al. describe their experience in the assessment of infants with seizure-like activity as one presenting clinical manifestation. Video Electroencephalography (vEEG) or standard EEG recordings were reviewed in detail. Epileptic seizures were rare, noted in 7.5% of their series. VEEG demonstrated disturbed non-rapid eye movement (REM) sleep with frequent arousal, jittery movements, and sleep myoclonus. VEEG would be useful prior to initiation of anti-convulsant medication in infants presenting clinically with seizure like activity. The final clinical science paper is offered by Devlin et al., who describe the successful work by her group to decrease pharmacologic treatment and length of stay for infants with NAS. They compared a new treatment algorithm to historic controls and found decreased need for treatment and length of hospital stay for infants with their new protocol, demonstrating significant financial savings.

## Future of NAS Research

Cole et al., gives us a review of the pharmacogenomics of NAS and how a better understanding of how genetics intersects with substance exposure can lead to novel methods of identifying at-risk patients and potentially better treatment practices. Finally, as we look to the future of NAS research, Westgate and Gomez-Pomar give us a framework to assess the value of the current and newly developing tools to describe the severity of NAS symptoms and suggest a formative modeling framework to judge these tools.

Unfortunately, in spite of many studies related to NAS, there is still a lot to learn so the knowledge can be applied to the prevention and management of infants with NAS. The problem of NAS requires a multifaceted approach that must be taken into consideration by those involved in its research and in the care of these children and their families and those involved in policy in shaping the landscape of opioid addiction.

## Author Contributions

All authors listed have made a substantial, direct and intellectual contribution to the work, and approved it for publication.

### Conflict of Interest

The authors declare that the research was conducted in the absence of any commercial or financial relationships that could be construed as a potential conflict of interest.
